# Measurement properties of instruments to assess pain in children and adolescents with cancer: a systematic review protocol

**DOI:** 10.1186/s13643-019-0945-4

**Published:** 2019-01-28

**Authors:** Erik A. H. Loeffen, Jennifer N. Stinson, Kathryn A. Birnie, Monique van Dijk, Ketan Kulkarni, Mienke Rijsdijk, Anna Font-Gonzalez, L. Lee Dupuis, Elvira C. van Dalen, Renée L. Mulder, Fiona Campbell, Wim J. E. Tissing, Marianne D. van de Wetering, Faith Gibson

**Affiliations:** 1 University of Groningen, University Medical Center Groningen, Beatrix Children’s Hospital, Department of Pediatric Oncology/Hematology, Groningen, The Netherlands; 2 Department of Anesthesia and Pain Medicine, The Hospital for Sick Children, University of Toronto, Toronto, ON Canada; 3 The Hospital for Sick Children, Toronto, ON Canada; 4 Department of Pediatric Surgery, Erasmus MC Sophia Children’s Hospital, Rotterdam, the Netherlands; 5 Division of Pediatric Hematology Oncology, Department of Pediatrics, IWK Health Centre, Halifax, Nova Scotia Canada; 6 Pain Clinic, Department of Anesthesiology, University Medical Center Utrecht, Utrecht, The Netherlands; 7 Department of Pediatric Oncology, Emma Children’s Hospital, Academic Medical Center, Amsterdam, the Netherlands; 8 Department of Pharmacy and Research Institute, The Hospital for Sick Children, University of Toronto, Toronto, ON Canada; 9 Leslie Dan Faculty of Pharmacy, University of Toronto, Toronto, ON Canada; 10 Centre for Outcomes and Experiences Research in Children’s Health, Illness, and Disability, Great Ormond Street Hospital for Children NHS Foundation Trust, London, UK; 11 University of Surrey, Guildford, UK

**Keywords:** Systematic review, Protocol, Pediatric oncology, Pain, Measurement instrument

## Abstract

**Background:**

Pain in children and adolescents with cancer has been identified as an area where many healthcare professionals seek guidance. This protocol details a systematic review whose aim is to explore current knowledge regarding measurement instruments to assess pain (and pain-related distress) in children and adolescents with cancer. After completion of the review, the information will be used in the development of a clinical practice guideline.

**Methods:**

We will search four electronic databases (MEDLINE via PubMed, CINAHL, PsycINFO and HaPI). Additional relevant studies will be identified by reference checking and expert consultation. All citations will be screened independently by two reviewers in a three-step approach: first selection based on title, second selection based on abstract, third selection based on full-text. Studies in children and adolescents with cancer that aimed to evaluate the clinimetric properties of an existing pain measurement instrument or to develop a new pain measurement instrument and that include at least one relevant outcome (reliability, validity, responsiveness, interpretability, clinical utility) are eligible for inclusion. For all steps of evidence selection, a detailed list with eligibility criteria will be determined a priori. Data extraction and quality assessment of included studies (according to the COnsensus-based Standards for the selection of health Measurement INstruments, COSMIN criteria) will be conducted independently by two authors.

**Discussion:**

This systematic review will provide an overview of the current literature regarding measurement instruments to assess pain in children and adolescents with cancer. This knowledge synthesis will be used to formulate recommendations for clinical practice. Also, by synthesizing existing evidence, knowledge gaps will be identified.

**Systematic review registration:**

PROSPERO CRD42017072879

**Electronic supplementary material:**

The online version of this article (10.1186/s13643-019-0945-4) contains supplementary material, which is available to authorized users.

## Background

The survival outcome for children with cancer has improved dramatically over the past 50 years [1]. In developed nations, the cure rates of childhood cancers now exceed 80%, whereas, in the 1960s, cure rates in children with cancer were the opposite: 20% [2, 3]. This remarkable increase can be attributed to a multitude of factors including the introduction of intensive treatment protocols and highly coordinated modern multidisciplinary care. However, these treatment modalities (mainly chemotherapy, radiotherapy, and surgery) also have clear undesirable side effects, both short- and long-term. Of these, pain is among the most prevalent and distressing [4, 5].

Besides improving survival outcome, an important focus in present day pediatric oncology is reducing morbidity and improving quality of life during and after treatment. This is referred to as supportive care, which the Multinational Association of Supportive Care in Cancer defined as “the prevention and management of the adverse effects of cancer and its treatment across the cancer continuum” [6]. As the field of supportive care is extremely broad, healthcare professionals often seek guidance on how to optimally assess and manage the undesirable effects of anti-cancer therapy [7]. To provide this guidance, the project “Towards evidence-based guidelines in childhood cancer” was initiated in 2014 [8].

As the first step in this project, we surveyed health care providers to determine the topic areas in which they wanted guidance. Pain assessment, evaluation (i.e., interpretation of the assessment to evaluate treatment effect), and management were identified as priorities [8, 9]. Regarding pain assessment, one of the great challenges in pediatric oncology is selecting measurement instruments that are age-appropriate, clinimetrically sound, and facilitate measurement of the various types of pain that children and adolescents with cancer experience.

In previous research, the focus has been placed upon synthesizing knowledge regarding pain measurement tools used with other populations. A systematic review on pain intensity assessment tools for adults concluded that numerical rating scales are applicable for unidimensional assessment of pain intensity in these patients [10]. Another recent systematic review focused on non-responsive adults and found that there are multiple observational pain assessment tools available for this population [11]. However, there are clear differences between children and adults, and there are various types of pain that children with cancer experience (e.g., neuropathic pain) that require specific guidance.

Thus, this systematic review will focus on assessment and evaluation of pain and pain-related distress and is an essential step in the development of a clinical practice guideline regarding pain in children and adolescents with cancer. For this guideline, the international guideline development panel consists of 46 members (including pediatric oncologists, nurses, anesthesiologists, pharmacologists, psychologists, and patient representatives) divided into a core group (leads, coordinators, advisors) and six working groups. Recommendations will be developed for assessment and evaluation of pain, as well as for pharmacological, physical, and psychological management of tumor-, treatment-, and procedure-related pain.

The aim of this systematic review is to answer the question: “Which are measurement instruments with strong clinimetric properties to assess pain in children and adolescents with cancer?” In line with the COnsensus-based Standards for the selection of health status Measurement INstruments (COSMIN) protocol for systematic reviews of measurement properties, we defined the:

1. Construct of interest, i.e., pain (and pain-related distress)
2. Population of interest, i.e., children and adolescents with cancer
3. Type of measurement instrument, i.e., self-report (using numbers or pictures/faces/colors), observer ratings, behavioral measurement, and multidimensional measurement
4. Measurement properties, i.e., reliability, validity, responsiveness, interpretability, and clinical utility

## Methods

The Preferred Reporting Items for Systematic Review and Meta-Analysis Protocols (PRISMA-P) statement has been followed in the development of this systematic review protocol [12]. The completed PRISMA-P checklist can be found in Additional file 1.

### Eligibility criteria

To be included in this review, studies need to meet the subjoined inclusion criteria. These are defined according to the COSMIN criteria:

#### Construct of interest

Studies that evaluate instruments that aim to measure the construct of pain (all types of pain). Distress is also taken into account, when related to pain assessment.

#### Population of interest

Studies that encompass children and adolescents with cancer, defined as (1) all participants < 25 years old or a median ≤ 16 years old or a mean ≤ 16 years old and (2) at least 75% of participants are diagnosed with cancer.

#### Type of measurement instrument

Studies that investigate a relevant measurement instrument, which differs per clinical question and is either self-reported (e.g., visual analog scale (VAS)) or a behavioral pain/distress measurement instrument (e.g., the COMFORT scale) [13, 14].

#### Measurement properties

At least one relevant outcome should be included. For all clinical questions, relevant outcomes are reliability, validity, responsiveness, interpretability, and clinical utility (as defined by COSMIN, see Table 1).

**Table 1:**
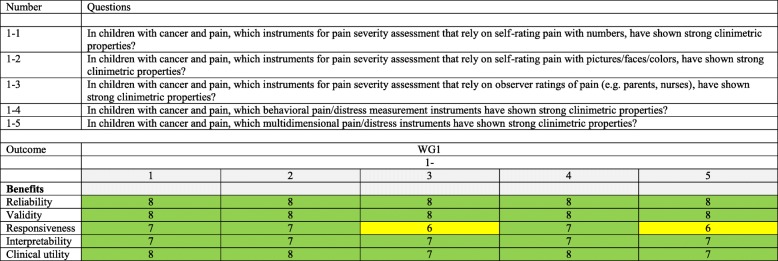
Overview of clinical questions and included outcomes per clinical question. Included outcomes differ per clinical question and are highlighted with a number/color. The number represents the median score of the outcome in the guideline development panel voting process. Outcomes with a score of 7–9 are considered critical to decision-making (highlighted green), outcomes with a score of 4–6 are considered important, but not critical to decision-making (highlighted yellow), and outcomes with a score of 1–3 are considered of low importance to decision-making (none in this case)

#### Eligible study types

Only primary, quantitative studies will be included. The sample size per study should be at least ten participants. Studies should clearly state their aim is to evaluate the clinimetric properties of an existing measurement instrument or to develop a new measurement instrument (this criterion is in line with COSMIN) [15]. Studies need to be published in a peer-reviewed medical journal, with a full text available in the English language.

### Literature searches

A search strategy has been developed in consultation with an information specialist of Cochrane Childhood Cancer with extensive expertise in systematic reviews. We will limit our results to publications in English, and a publication date filter will not be imposed. The search consists of four search strategies combined with the ‘AND’ Boolean operator, focusing on:

1. Children, with free text terms such as infan* and child*, and MeSH headings such as pediatrics[mh]
2. Childhood cancer types, with free text terms such as osteosarcom* and meningiom*, and MeSH headings such as (leukemia, lymphocytic, acute[mh])
3. Pain, with free text terms such as neuropath* and ache, and MeSH headings such as pain[mh]
4. Measurement properties, with free text terms such as validi* and test-retest*, and MeSH headings such as psychometrics[mh] [16, 17]

The searches will be conducted in four electronic databases, i.e., the Medical Literature Analysis and Retrieval System Online (MEDLINE, via PubMed), the Cumulative Index to Nursing and Allied Health Literature (CINAHL), the Psychological Information Database (PsycINFO), and the Health and Psychosocial Instruments Database (HaPI). As an example, the full search strategy that will be used in PubMed/MEDLINE can be found in Table 2. For identification of additional studies that were not included in this search, we will check all references of included studies (backwards citation chasing) and all studies that referenced the included studies (forward citation chasing). Also, we will consult experts for missing studies that meet the inclusion criteria. In addition, to identify promising ongoing and recently completed studies, we will hand search (1) conference proceedings of the International Society on Pediatric Oncology (SIOP) (2012 to 2017), the American Society of Clinical Oncology (ASCO) (2012 to 2017), the American Academy of Pediatrics (AAP) (2012 to 2017), the International Symposium on Pediatric Pain (ISPP) (2012 to 2017), the American Academy of Pain Medicine (AAPM) (2012 to 2017), the European Pain Federation (EFIC) (2012 to 2017), and the Multinational Association for Supportive Care in Cancer (MASCC) (2012 to 2017), dependent on availability and (2) the trial registers of the National Institute of Health (NIH) Register for ongoing trials and the World Health Organization (WHO) International Clinical Trials Registry Platform (ICTRP).

**Table 2:** Search strategy for PubMed/MEDLINE

#	Search history	Results (March 22, 2017)	Explanation
#1	((leukemia OR leukemi* OR leukaemi* OR (childhood ALL) OR AML OR lymphoma OR lymphom* OR hodgkin OR hodgkin* OR T-cell OR B-cell OR non-hodgkin OR sarcoma OR sarcom* OR sarcoma, Ewing’s OR Ewing* OR osteosarcoma OR osteosarcom* OR wilms tumor OR wilms* OR nephroblastom* OR neuroblastoma OR neuroblastom* OR rhabdomyosarcoma OR rhabdomyosarcom* OR teratoma OR teratom* OR hepatoma OR hepatom* OR hepatoblastoma OR hepatoblastom* OR PNET OR medulloblastoma OR medulloblastom* OR PNET* OR neuroectodermal tumors, primitive OR retinoblastoma OR retinoblastom* OR meningioma OR meningiom* OR glioma OR gliom*) OR (pediatric oncology OR paediatric oncology) OR (childhood cancer OR childhood tumor OR childhood tumors)) OR (brain tumor* OR brain tumour* OR brain neoplasms OR central nervous system neoplasm OR central nervous system neoplasms OR central nervous system tumor* OR central nervous system tumour* OR brain cancer* OR brain neoplasm* OR intracranial neoplasm*) OR (leukemia, lymphocytic, acute[mh]) OR (leukemia, lymphocytic, acute*)	1.641.231	Search filter for childhood cancer (from Cochrane Childhood Cancer)
#2	Infan* OR toddler* OR minors OR minors* OR boy OR boys OR boyfriend OR boyhood OR girl* OR kid OR kids OR child OR child* OR children* OR schoolchild* OR schoolchild OR school child[tiab] OR school child*[tiab] OR adolescen* OR juvenil* OR youth* OR teen* OR under*age* OR pubescen* OR pediatrics[mh] OR pediatric* OR paediatric* OR peadiatric* OR school[tiab] OR school*[tiab]	4.334.396	Search filter for children (from Cochrane Childhood Cancer)
#3	pain[mh] OR acute pain[mh] OR chronic pain[mh] OR pain OR ache OR agony OR hyperalgesia[mh] OR allodynia OR analgesia OR distress OR headache OR hurt[tiab] OR hyperesthesia OR hyperaesthesia OR myalgia OR neuralgia OR neuropath* OR polyneuropathy OR painful[tiab]	1.026.012	Search filter for pain (developed in consultation with the information specialist from the Cochrane Childhood Cancer)
#4	Validation Studies[pt] OR “psychometrics”[MeSH] OR psychometr*[tiab] OR clinimetr*[tw] OR clinometr*[tw] OR reproducibi*[tiab] OR reliabi*[tiab] OR unreliabi*[tiab] OR validi*[tiab] OR valida*[tiab] OR “internal consistency”[tiab] OR precision[tiab] OR imprecision[tiab] OR test–retest[tiab] OR repeatabi*[tiab] OR ((multitrait[tiab] AND scaling[tiab]) AND (analysis[tiab] OR analyses[tiab])) OR item discriminant[tiab] OR sensitivi*[tiab] OR specifici* OR ((minimal[tiab] OR minimally[tiab]) AND (clinical[tiab] OR clinically[tiab]) AND (important[tiab] OR significant[tiab] OR detectable[tiab]) AND (change[tiab] OR difference[tiab])) OR (small* AND (real[tiab] OR detectable[tiab]) AND (change[tiab] OR difference[tiab])) OR pain measurement[mh]	2.068.426	Search filter measurement properties (developed in consultation with the information specialist from the Cochrane Childhood Cancer)
#5	#1 AND #2 AND #3 AND #4	1.804	
#6	#5, filters: Humans; English	1.498	

All citations that will be identified in the database searches will be imported in EndNote X8 (Clarivate Analytics, Philadelphia, PA, USA) after which duplicates will be removed. For appraisal of the citations, we will use exported Microsoft Excel files (Microsoft, Redmond, WA, USA).

### Study selection

**Fig. 1:**
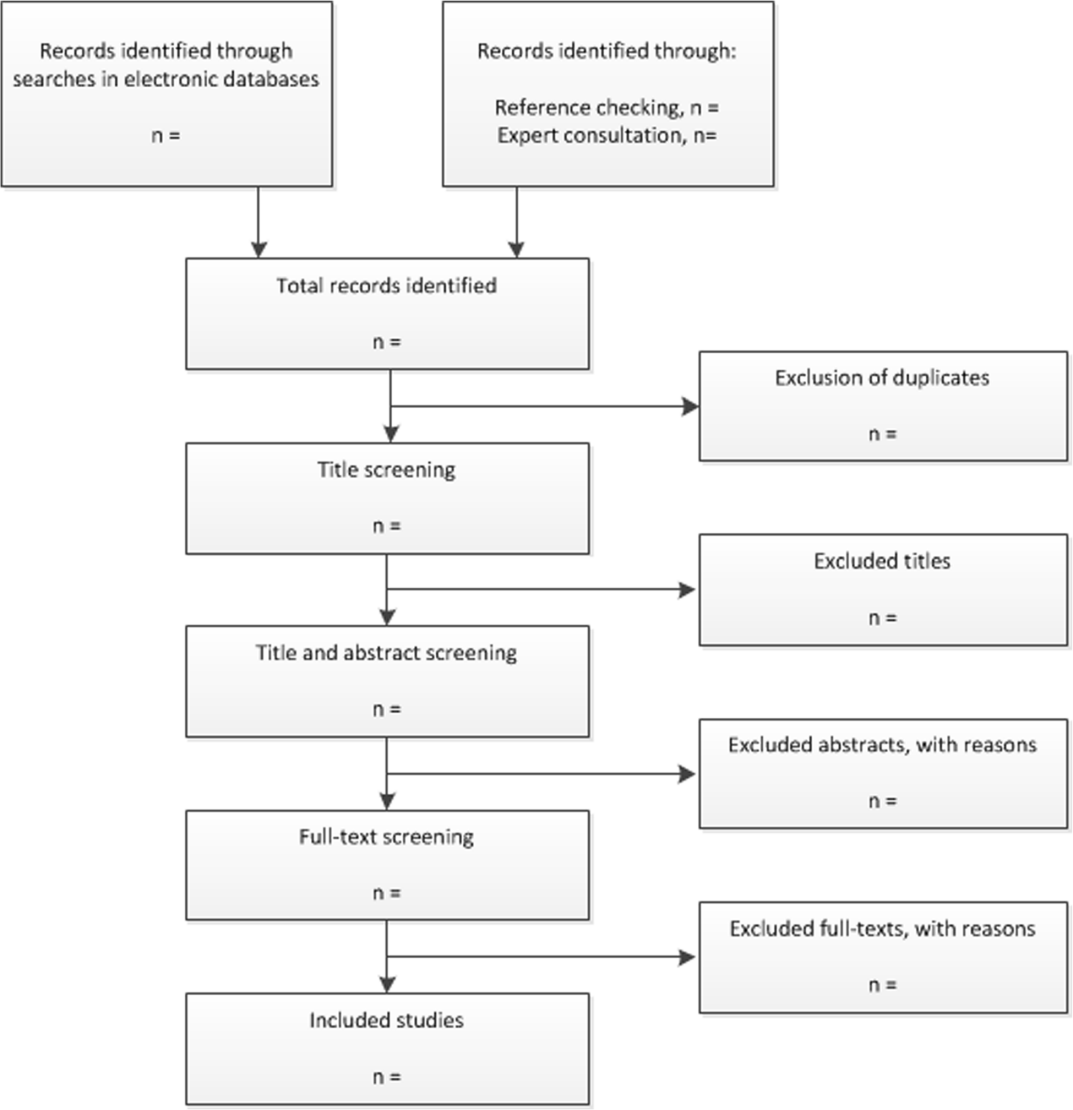
Flow diagram of the study selection process

We have developed an algorithm that will be followed in the phase of evidence appraisal (see Fig. 1). The study selection will consist of three phases.

#### Title selection

As we expect a large total number of citations, the first round will consist of selection based on titles only. A recent study that compared screening titles alone versus titles and abstracts concluded that screening on title alone may be more effective [18]. The entire selection will be performed in duplicate by two independent reviewers (with extensive experience in both pediatric oncology and in systematic reviews, 1: EL, 2: WT). This serves to exclude studies that are obviously irrelevant for this review. A conservative approach to inclusion will be employed to ensure relevant titles are not excluded. All citations classified as “include” by at least one reviewer will be included, even if the second reviewer classified the citation as “exclude” (no discussion will be held). It should be noted that this conservative approach is only applied during the title selection phase. During abstract and full-text selection, all discrepancies will be discussed in detail and resolved by consensus.

To pilot the title selection process, two reviewers will appraise the first 250 citations. If there is an agreement below 85% percent, the title selection criteria will be optimized and the pilot will be repeated on the next 250 citations. This continues until the agreement is at least 85%.

#### Abstract selection

The second round will consist of selection based on title and abstract. The entire selection will be performed in duplicate by two independent reviewers (1: EL, 2: divided among JS, KB, MD, KK, MR, MW, and FG). A pilot will be performed with the first 100 citations in a similar fashion as with the title selection pilot. Hereafter, discrepancies will be resolved by consensus, and if this is not reached, a third reviewer will be consulted.

#### Full-text selection

In the final selection round, two independent reviewers (the same as in the second round) will perform the selection of the full texts. Discrepancies will be resolved by consensus, and if this is not reached, a third reviewer will be consulted.

### Data extraction

Two reviewers will independently extract data from each included study using a data abstraction form designed for this systematic review (see Additional file 1). This form will be pilot-tested by two reviewers with a representable sample of studies and, if necessary, optimized according to the reviewers’ comments. The data extraction form differs slightly for each clinical question, but includes at minimum (1) general study information (i.e., first author, title, journal, year, funding source), (2) study design characteristics (i.e., study design, setting, duration, sample size), (3) participant characteristics (i.e., age, gender, diagnosis, age at diagnosis, type of pain), (4) measurement instrument characteristics (i.e., instrument used, measurement method/dimensions), (5) outcome characteristics (i.e., included outcomes, outcome values), and (6) additional information (differs per study, at the discretion of the reviewer). Reviewers will discuss the completed forms, and discrepancies will be resolved by consensus. If consensus is not reached, a third reviewer will be consulted. In case of missing information, the authors of the included study will be contacted in an effort to obtain this information.

### Quality assessment

Two reviewers will independently assess the quality of the included studies, according to the COSMIN checklist for assessing methodological quality of studies on measurement properties [15, 19]. This checklist is purpose-built for the evaluation of measurement properties and takes a three-tier approach. For multi-item measures, it is first determined if the statistical methods are based on the classical test theory (CCT) or on the item response theory (IRT); in the case of the latter, a separate quality checklist is included. Second, a checklist is completed for each measurement property evaluated in each included paper to evaluate if standards for good methodological quality are met. Third and last, a checklist regarding the generalizability of each outcome is completed. Discrepancies between reviewers will be resolved in the same manner as described for data extraction.

The COSMIN 4-point checklist scoring system will result in a score per included outcome for each study that can either be “excellent” (evidence that the methodological quality is adequate), “good” (assumed that the methodological quality is adequate, but relevant information is not reported), “fair” (doubtful whether the methodological quality is adequate), or “poor” (evidence that the methodological quality is not adequate). This serves the purpose of identifying the measurement tool with the strongest clinimetric properties.

Regarding publication bias, we will seek to identify cues that imply this bias, e.g., identification of merely studies that report measurement instruments with strong clinimetric properties. A prospective registration database, as there is for randomized controlled trials, does not exist for measurement properties studies, which makes identifying publication bias particularly hard in this area of research. Also, there is no established methodology for this, e.g., as there is for a meta-analysis with a funnel plot. Therefore, we will comment upon this in a qualitative manner.

### Synthesis of results

Firstly, we will prepare a table of characteristics of included studies where information regarding design, sample, measurement instrument(s), and outcome(s) will be presented. Outcome tables will be prepared to cover each measurement instrument (e.g., the Comfort Behavior Scale) and each outcome construct (e.g., self-reported pain intensity) separately. In addition, an outcome table will be prepared which categorizes instruments according to age group, i.e., 0–0.99 years (infants), 1–2.99 years (children not able to speak or read), 3–6.99 years (children able to speak, not able to read), 7–12.99 years (children able to speak and read), and 12–18 years (teenagers). If the identified evidence leads to the conclusion that differently defined age groups are more appropriate, we will reconsider the definition of the age groups.

To provide a comprehensive overview, we will develop a quality matrix. Herein, we will list all included measurement instruments and the identified information on purpose (self- or observer-report, type of pain), number of studies, population/age group, and per outcome COSMIN quality score (four-level). This will allow the reader to quickly identify the appropriate measurement instrument with the strongest clinimetric properties.

In addition, we will present a narrative synthesis discussing our findings.

## Discussion

In this protocol, we describe our approach for a systematic review regarding measurement properties of instruments to assess pain and pain-related distress in children and adolescents with cancer. This overview is much needed for multiple reasons.

First, there is a clear difference between adults and children. Second, besides this difference, there is also a great difference between children across ages (e.g., a 15-year-old and a 3-year-old). Children go through various developmental stages which lead to great variation in this population in terms of abilities in verbal communication, pain experience, and associative thinking, which will determine the appropriateness of individual instruments within specific age groups [20]. Although self-report is often regarded as the ideal method for assessing pain, in young children, this is not always possible. Therefore, these populations have to rely on the people around them (family members but also healthcare professionals) to notice and act upon their pain. This underlines the importance of having clinimetrically sound measurement instruments not just for self-report, but also for proxy report. Third, children and adolescents with cancer have various specific types of pain which are often related to their disease (e.g., bone pain due to metastases) or treatment (e.g., procedural pain, vinca alkaloid-associated neuropathic pain). Fourth, pain severity measurement is the critical first step to determine the need for treatment and to evaluate the success of treatment. When pain is not measured, it is often undertreated [21]. All of these reasons call for age-specific measurement instruments with strong clinimetric properties to assess pain in children and adolescents with cancer.

Currently, there is great interest in reducing the side-effects of cancer treatment in children and adolescents, to reduce morbidity and improve quality of life. Pain is frequently experienced by children and adolescents with cancer and carries with it a great physical and psychological burden [4]. It is known that not acknowledging and treating pain can have a detrimental effect on quality of life [22]. Therefore, it is of utmost importance that healthcare professionals have access to the available evidence and evidence-based recommendations for care.

To address these needs, we have initiated a clinical practice guideline development project that focuses on formulating recommendations on the assessment and treatment of pain in children and adolescents with cancer. This systematic review of the literature describing pain measurement instruments is the first step towards formulating these recommendations.

## Additional file

Additional file 1: PRISMA-P 2015 Checklist – Loeffen et al. - Measurement properties of instruments to assess pain in children with cancer: A systematic review protocol. (DOCX 38 kb)

## Abbreviations

CINAHL: Cumulative Index to Nursing and Allied Health Literature
COSMIN: COnsensus-based Standards for the selection of health Measurement INstruments
EMBASE: Excerpta Medica Database
GRADE: Grading of Recommendations Assessment, Development and Evaluation
HaPI: Health and Psychosocial Instruments Database
MEDLINE: Medical Literature Analysis and Retrieval System Online
PRISMA-P: Preferred Reporting Items for Systematic Review and Meta-Analysis Protocols
PROSPERO: International prospective register of systematic reviews
PsycINFO: Psychological Information Database
VAS: Visual analog scale
